# *N* ^6^-methyladenosine enhances post-transcriptional gene regulation by microRNAs

**DOI:** 10.1093/bioadv/vbab046

**Published:** 2022-01-18

**Authors:** Shaveta Kanoria, William A Rennie, Charles Steven Carmack, Jun Lu, Ye Ding

**Affiliations:** 1 Wadsworth Center, New York State Department of Health, Center for Medical Science, Albany, NY 12208, USA; 2 Department of Genetics and Yale Stem Cell Center, Yale University, New Haven, CT 06520, USA

## Abstract

**Motivation:**

*N* ^6^-methyladenosine (m^6^A) is the most prevalent modification in eukaryotic messenger RNAs. MicroRNAs (miRNAs) are abundant post-transcriptional regulators of gene expression. Correlation between m^6^A and miRNA-targeting sites has been reported to suggest possible involvement of m^6^A in miRNA-mediated gene regulation. However, it is unknown what the regulatory effects might be. In this study, we performed comprehensive analyses of high-throughput data on m^6^A and miRNA target binding and regulation.

**Results:**

We found that the level of miRNA-mediated target suppression is significantly enhanced when m^6^A is present on target mRNAs. The evolutionary conservation for miRNA-binding sites with m^6^A modification is significantly higher than that for miRNA-binding sites without modification. These findings suggest functional significance of m^6^A modification in post-transcriptional gene regulation by miRNAs. We also found that methylated targets have more stable structure than non-methylated targets, as indicated by significantly higher GC content. Furthermore, miRNA-binding sites that can be potentially methylated are significantly less accessible without methylation than those that do not possess potential methylation sites. Since either RNA-binding proteins or m^6^A modification by itself can destabilize RNA structure, we propose a model in which m^6^A alters local target secondary structure to increase accessibility for efficient binding by Argonaute proteins, leading to enhanced miRNA-mediated regulation.

**Availability and implementation:**

N/A.

## 1 Introduction

Methylation of the N^6^ position of adenosine (m^6^A) is the most prevalent modification in eukaryotic mRNAs (Liu and Pan, 2016; Meyer and Jaffrey, 2017; Roundtree *et al.*, 2017). m^6^A methylation is mediated by methyltransferases (m^6^A writers) that include methyltransferase-like 3 (METTL3) and METTL14. The modification can be erased by RNA demethylases (m^6^A erasers), such as ALKBH5 (Zheng *et al.*, 2013). Recognition of m^6^A by m^6^A-binding proteins (m^6^A readers) is a major mechanism for effects of the modification (Meyer and Jaffrey, 2017). Transcriptome-wide single-nucleotide resolution mapping has revealed that m^6^A sites are enriched in the 3′-untranslated regions (3′-UTRs) and near stop codon, and that there is an association or correlation between m^6^A and predicted microRNA (miRNAs)-binding sites on mRNAs (Das Mandal and Ray, 2021; Ke *et al.*, 2015; Meyer *et al.*, 2012). m^6^A modification plays important regulatory roles in a variety of fundamental cellular processes. Examples are regulation of pre-mRNA splicing (Louloupi *et al.*, 2018), control of cell fate transition (Batista *et al.*, 2014), regulation of mRNA stability (Wang *et al.*, 2014), processing of primary miRNAs (Alarcon *et al.*, 2015) and cell reprogramming (Chen *et al.*, 2015). Emerging evidence also suggests an association between m^6^A modification and cancer progression (Chen *et al.*, 2019). N^6^-adenosine methylation in some miRNAs has been observed (Berulava *et al.*, 2015; Ke *et al.*, 2015). It was recently reported that miRNA-mediated loss of m^6^A increases nascent translation in glioblastoma (Zepecki *et al.*, 2021).

miRNAs are an abundant class of small non-coding RNAs of about ∼22 nt that have been found in plants, animals and some viruses (Ambros, 2004; Bartel, 2004) A mature miRNA contained in an RNA-induced silencing complex hybridizes to partially complementary sequences typically in the 3'-UTRs of the target mRNAs, leading to translational repression and/or mRNA degradation of the target mRNA. miRNAs play important roles in development, differentiation, apoptosis and proliferation (Bartel, 2004; Harfe, 2005). Moreover, mis-regulation in miRNA activity has been found to be associated with cancer and other human diseases (Erson and Petty, 2008; Esau and Monia, 2007).

Computational identification of target-binding sites has been primarily based on the seed, a key sequence feature for miRNA targeting (Lewis *et al.*, 2005). In addition, the importance of target structural accessibility for miRNA targeting has been established (Long *et al.*, 2007). With the development of the cross-linking immunoprecipitation (CLIP) technique (Chi *et al.*, 2009; Hafner *et al.*, 2010), it has become possible to identify short Argonaute (AGO)-crosslinked sequences containing miRNA-binding sites. By utilizing such data and a comprehensive list of sequence, thermodynamic and target structure features, models and software were developed for statistical prediction of both seed and seedless-binding sites (Liu *et al.*, 2013; Rennie *et al.*, 2014). Through the addition of a ligation step in the CLIP framework, the CLASH method allows direct observation of miRNA: target interactions (Helwak *et al.*, 2013). Therefore, the CLASH study presented a high-quality dataset of high-confidence miRNA-binding sites for analysis.

Since m^6^A methylation is enriched in the 3'-UTRs, the primary target regions of miRNAs, the question arises whether there is any regulatory impact of m^6^A modification on post-transcriptional regulation by miRNAs beyond the reported correlation between the methylation and miRNA-targeting sites (Das Mandal and Ray, 2021). To investigate this, we utilized multiple high-throughput data on m^6^A in human transcriptome (Sun *et al.*, 2016), and miRNA target binding and regulation (Baek *et al.*, 2008; Helwak *et al.*, 2013). We also performed conservation analysis as well as structural accessibility analysis for interpretation of our findings.

## 2 Methods

### 2.1 m^6^A modification data

We downloaded the data of m^6^A modification sites in mRNAs from the RMBase database (Sun *et al.*, 2016). A total of 140 574 single-nucleotide modification sites for human mRNAs and 84 539 for mouse mRNAs were available from the database and were all included in our analyses. The m^6^A modifications in RMBase database were all assembled from various studies involving high-throughput m6A-seq experiments. This method integrates immunoprecipitation of methylated, randomly fragmented RNA using a highly specific anti-m^6^A antibody to obtain an enriched population of modified fragments and massively parallel sequencing, resulting in mapping of this modification throughout the transcriptome.

### 2.2 Classification of miRNA targets and binding sites

In this study, a transcript is termed as m^6^A+ if it possesses at least one m^6^A modification site, and m^6^A− otherwise. Similarly, a miRNA-binding site is m^6^A+ if it overlaps with at least one m^6^A methylation site, and m^6^A− otherwise.

### 2.3 miRNA targeting data

For regulatory effects of mRNA targeting, we used high-throughput mRNA expression data from overexpression of human miR-1, miR-181 and miR-124, and knockout of mouse miR-223 (Baek *et al.*, 2008). Our dataset selection criteria were: (i) data reported the gene expression consequences after perturbing the expression of a single miRNA (overexpression or KO) in either human or mouse cells; (ii) microarray or RNAseq studies were performed before and after miRNA manipulation to allow for estimates of mRNA fold changes; and (iii) accurate assembly of seed targets among all mRNAs in the experimental system was provided in the publication. For human miRNA overexpression, there were 19 864 expression measurements from microarray. For miR-223 knockout, there were 20 334 expression measurements. Protein expression data were also available from this study. However, the size of this protein dataset is only about 15–20% of the mRNA dataset. For example, for miR-124, only 13 m^6^A− transcripts with either an 8mer or a 7mer seed site have ≥6 independent protein measurements, a consideration in the previous data analysis (Baek *et al.*, 2008). For our analyses, the proteomic data were too limited in size to be included. On the other hand, it has been shown that, to a great extent, changes in mRNA levels reflect the impact of mammalian miRNAs on gene expression (Guo *et al.*, 2010). For these reasons, our study focused on the analyses of the large mRNA data.

The second dataset for miRNA binding was based on the CLASH method to reveal high-confidence miRNA: target interactions (Helwak *et al.*, 2013), which allows for accurate identification of miRNA-binding sites on target mRNAs, albeit with unknown regulatory impact. This study identified ∼18 500 miRNA: target interactions for 7390 transcripts and 399 miRNAs. A majority of the interactions did not have canonical seed base pairing (i.e. binding sites were seedless).

### 2.4 Identification of miRNA-binding sites

For CLASH data, the STarMir program (Rennie *et al.*, 2014) was used to identify both seed and seedless binding sites in the target regions within the CLASH chimeras. STarMir incorporates the RNAhybrid program in model-based predictions (Liu *et al.*, 2013; Rehmsmeier *et al.*, 2004). For mRNAs with expression measurements from overexpression of human miR-1, miR-181 and miR-124, and knockout of mouse miR-223, the STarMir program was used for prediction of miRNA-binding sites.

### 2.5 Computation of site conservation, structural accessibility and statistical significance

For each miRNA-binding site, we computed a site conservation score as the average of individual nucleotide conservation scores available from the UCSC genome browser (Kent *et al.*, 2002; Siepel *et al.*, 2005). For analysis on target site accessibility, we computed ΔG_total_, a miRNA-target-hybridization-model-based quantitative measure of local structural accessibility (Long *et al.*, 2007). Because the calculation was based on RNA thermodynamics for unmodified nucleotides (Mathews *et al.*, 1999), the analysis only revealed the degree of accessibility before any potential effects by m^6^A methylation. To assess the statistical significance of pairwise distributional comparisons, the *P*-values from Kolmogorov–Smirnov tests were reported.

## 3 Results

### 3.1 Higher levels of regulation for targets with m^6^A methylation

**Fig. 1.**
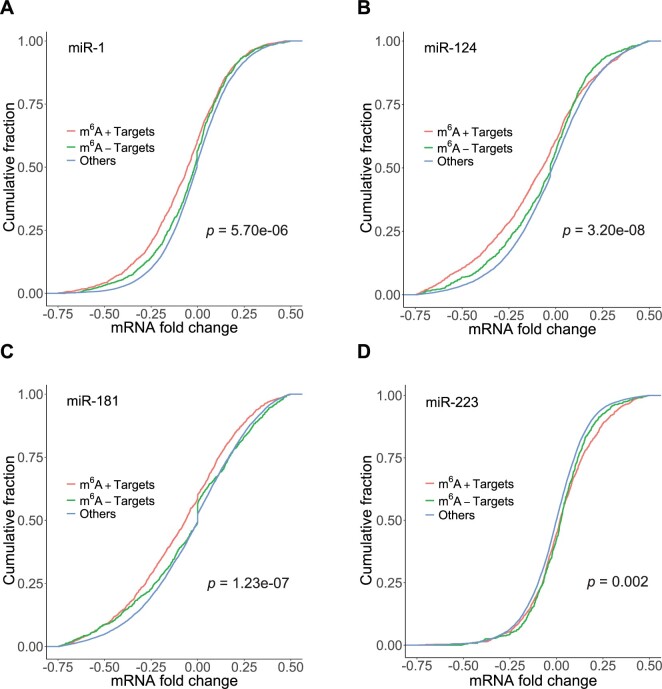
Impact of m6A methylation on miRNA regulation. Comparison of cumulative distributions of mRNA fold changes (in log_2_ scale) for m^6^A+ targets (red), m^6^A− targets (green) and ‘Others’ (blue), in response to (**A**) miR-1 overexpression (number of targets is 2879), with *P*-values of 5.70e-06 for red versus green, <2.2e-16 for red versus blue and 0.011 for green versus blue; (**B**) miR-124 overexpression (number of targets is 3202), with *P*-values of 3.20e-08 for red versus green, <2.2e-16 for red versus blue and 4.71e-09 for green versus blue; (**C**) miR-181 overexpression (number of targets is 3517), with *P*-values of 1.23e-07 for red versus green, <2.2e-16 for red versus blue and 2.78e-06 for green versus blue; and (**D**) miR-223 knockdown (number of targets is 2268), with *P*-values of 0.002 for red versus green (among the up-regulated), 2.687e-14 for red versus blue and 6.22e-06 for green versus blue

A previous study (Baek *et al.*, 2008) presented data of mRNA fold changes (in log2 scale) for target mRNAs with an 8mer or a 7mer (A1 or m8) binding site, showing that such sites are more effective for miRNA targeting. Thus, to examine whether m^6^A methylation on mRNAs has any effects on regulation by miRNAs, we focused our analysis on these targets. Among 2879 targets for human miR-1, 1519 were m^6^A+ and 1360 were m^6^A−. For targets of human miR-124, 1841 were m^6^A+ and 1361 were m^6^A−. For human miR-181, 1920 targets were m^6^A+ and 1597 were m^6^A−. For targets of mouse miR-223, 1276 were m^6^A+ and 922 were m^6^A−. For miR-1 overexpression, the levels of down-regulation for m^6^A+ targets were significantly higher than m^6^A− targets (Fig. 1A; *P*-value of 5.70e-06). Similarly, for overexpression of miR-124 (Fig. 1B; *P*-value of 3.20e-08) or miR-181 (Fig. 1C; *P*-value of 1.23e-07), m^6^A+ targets were down-regulated to a greater extent than m^6^A− targets. For miR-223 knockout, for up-regulated targets (i.e. log_2_-fold change >0), the levels of up-regulation for m^6^A+ targets were significantly higher than m^6^A− targets (Fig. 1D; *P*-value of 0.002). All together, these results indicate that m^6^A modification on target transcripts significantly enhances effects of regulation by miRNAs.

Also plotted in Figure 1 is the empirical cumulative distribution for ‘Others’. This group was formed from the whole set of mRNAs by removing 7-mer, 8-mer targets and 6mer targets with a probability of 0.5 or higher as predicted by STarMir. The probability is a model-based measure of confidence that a predicted site is bound by AGO (Liu *et al.*, 2013). Thus, the group does not include higher confidence seed targets; however, it could contain other targets harboring only seedless sites. The levels of regulation for this group were significantly less than either the m^6^A+ targets (*P*-value <2.2e-16 for miR-1, miR-124, miR-181 and 2.687e-14 for miR-223), or the m^6^A− targets (*P*-value of 0.011, 4.71e-09, 2.78e-06 and 6.22e-06, for miR-1, miR-124, miR-181 and miR-223, respectively).

### 3.2 Higher conservation for m^6^A+ miRNA sites

**Fig. 2.**
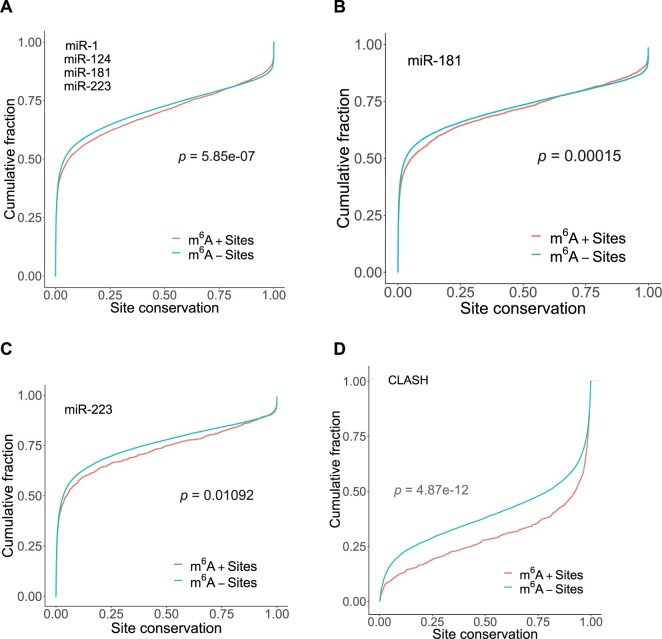
m^6^A+ miRNA sites are more conserved. Comparison of cumulative distributions of conservation scores between m^6^A+ binding site (red) and m^6^A− binding sites (blue), for (**A**) pooled miRNA-binding sites for miR-1 (928 m^6^A+ sites, 28 876 m^6^A− sites), miR-124 (1715 m^6^A+ sites, 51 467 m^6^A− sites), miR-181 (2460 m^6^A+ sites, 52 900 m^6^A− sites) and miR-223 (1283 m^6^A+ sites, 48 989 m^6^A− sites), with a *P*-value of 5.85e-07; (**B**) miR-181, with a *P*-value of 0.0001492; (**C**) miR-223, with a *P*-value of 0.01092; and (**D**) miRNA sites identified from CLASH (number of miRNAs is 399; total number of sites is 18 500), with a *P*-value of 4.87e-12

We investigated whether the presence of m^6^A has any effects on the conservation of miRNA-binding sites. For miR-1, miR-124, miR-181 and miR-223, we predicted miRNA-binding sites using the STarMir program (Rennie *et al.*, 2014) to identify the nucleotide positions of both seed and seedless sites in all mRNAs. Position information for seed sites was not provided in the previous study (Baek *et al.*, 2008). Pooling the four miRNAs to achieve greater statistical power, we found that the m^6^A+ binding sites have significantly higher conservation than m^6^A− binding sites (Fig. 2A, *P*-value of 5.85e-07). For the individual miRNAs, significance was observed for miR-1 (*P*-value of 0.005688), miR-181 (*P*-value of 0.0001492, Fig. 2B) and miR-223 (*P*-value of 0.01092, Fig. 2C). There was a higher conservation for miR-124, but it was insignificant at 0.05 level (*P*-value of 0.1387).

For CLASH data on 399 miRNAs and 7390 transcripts, the conservation for m^6^A+ miRNA-binding sites was also significantly higher than for m^6^A− miRNA-binding sites (Fig. 2D; *P*-value of 4.87e-12). Higher conservation suggests functional relevance of m^6^A in gene regulation by miRNAs.

### 3.3 Analyses of potential confounding factors

#### 3.3.1 Distance from m^6^A site to miRNA-binding site

To investigate whether the distance from the site of m^6^A modification to the site of miRNA binding has any effect on miRNA-mediated regulation, we divided m^6^A+ targets into two subsets: one with distances under 100 nt (‘shorter distance subset’), and the other with distances of 100 nt or greater (‘longer distance subset’). In cases having multiple m^6^A sites on the same target 3′-UTR, the one with the shortest distance was used. The nucleotide positions of the 7mer or 8mer binding sites were identified using the RNAhybrid program (Rehmsmeier *et al.*, 2004). In cases with both a 7mer and an 8mer site present on the same 3′-UTR, the 8mer site was used due to its generally stronger regulation (Bartel, 2004); in cases of multiple 7mer sites or multiple 8mer sites, the one with the stronger hybridization energy was used to represent the corresponding site type.

For miR-1 and miR-124, the shorter distance target subset had a significantly higher level of regulation than the longer distance target subset (*P*-value of 0.045 for miR-1 and 0.049 for miR-124). However, this effect was not observed for miR-181 (*P*-value of 0.138) or miR-223 (*P*-value of 0.664). These observations suggest that for some miRNAs, a shorter distance from the m^6^A site to the miRNA-binding site exerts a greater level of regulatory enhancement.

#### 3.3.2 Target 3′-UTR length

We compared the lengths of 3′-UTRs between m^6^A+ and m^6^A− targets. For all four miRNAs, we found that m^6^A+ targets had significantly longer 3′-UTRs as compared to m^6^A− targets (*P*-values are 8.128e-10 for miR-1, 5.917e-13 for miR-124 and <2.2e-16 for miR-181 and miR-223).

To control for 3′-UTR length in a reanalysis, we set up length bin of 500 nt, i.e. (0 nt, 500 nt), (500 nt, 1000 nt)… (4500 nt, 5000 nt) and (5000 nt, +∞). For each bin, with a preset common sampling size, we randomly sampled UTRs for m^6^A+ targets and m^6^A− targets. For example, in the human data, for bin (500 nt, 1000 nt), there were 5505 m^6^A+ targets and 3232 m^6^A− targets. We set the sampling size at 3000. Because each bin was equally represented by m^6^A+ targets and m^6^A− targets, the cumulative distributions of the 3′-UTR length for the sampled m^6^A+ targets and the sampled m^6^A− targets were nearly identical, with insignificant *P*-values (data not shown). For the sampled targets, we repeated the previous analysis. We found that, for miR-181 and miR-124, the level of regulation was higher for the sampled m^6^A+ targets as compared to the sampled m^6^A− targets (*P*-value of 3.169e-06 for miR-181 and 4.203e-07 for miR-124). For miR-1 and miR-223, however, there was no significant enhancement by m^6^A (*P*-value of 0.539 for miR-1 and 0.3548 for miR-223). These findings suggest that, for some miRNAs, longer 3′-UTR has a positive effect on the enhancement of regulation.

#### 3.3.3 miRNA-binding location within 3′-UTR

We next examined whether there was a difference in the location of miRNA-binding sites between the m^6^A+ targets and the m^6^A− targets. For the *k*th nucleotide in a 3′-UTR of *n* nucleotides, the relative location within the 3′-UTR was (*k*/*n*) ×100%. For miR-1 and miR-124, miRNA-binding sites for the m^6^A+ targets were located significantly farther away from the start of 3′-UTR, in comparison to the m^6^A− targets (*P*-value of 0.003285 for miR-1 and 0.02981 for miR-124). For miR-181 and miR-223, the difference in location was not significant (*P*-value of 0.6407 for miR-181 and 0.88 for miR-223).

#### 3.3.4 Target basal expression

Using expression data for wild-type cells (i.e. control sample for mRNA overexpression or KO), we found there was no significant difference in basal expression levels between m^6^A+ targets and m^6^A− targets for all three human datasets (*P*-value of 0.5618 for miR-1 control sample; 0.8651 for miR-124 control sample; and 0.06 for miR-181 control sample). For the mouse miR-223 control sample, however, there was a significantly higher basal expression for the m^6^A+ targets as compared to the m^6^A− targets (*P*-value of 8.164e-10).

#### 3.3.5 Proportion of 7mer or 8mer binding sites

For each miRNA, we compared the proportion of 7mer or 8mer binding sites between the m^6^A+ targets and the m^6^A− targets. We did not observe an appreciable difference (data not shown).

#### 3.3.6 Proximal m^6^A sites within 200 nt of miRNA-binding sites

In this study, a m^6^A+ site required an overlap between the m^6^A site and a miRNA-binding site. However, it is of interest to consider proximal m^6^A sites that are outside the miRNA-binding site. It has been reported that 60% of m^6^A sites are within 200 nt of miRNA-binding sites (Ke *et al.*, 2015). For the m^6^A+ targets used in the analysis for Figure 1, we removed those with m^6^A sites overlapping with miRNA-binding sites as well as those with m^6^A sites located outside the 200 nt window. The remaining targets were compared with the m^6^A− targets. We found that there was significant regulatory enhancement for miR-1 and miR-124 (*P*-value of 0.0001369 and 0.001936, respectively). This suggests that in some cases, proximal m^6^A sites can have a positive effect on gene regulation. The effect may depend on other factors, such as structural accessibility.

In summary, of the factors examined above, we could not identify one that could potentially explain the findings in Figure 1 for all four miRNAs. A remaining factor to be examined was the GC content, which is reported in the next subsection, as it has implications for RNA structural stability.

### 3.4 Higher GC content for methylated target and lower structural accessibility for m6A+ binding sites

**Fig. 3.**
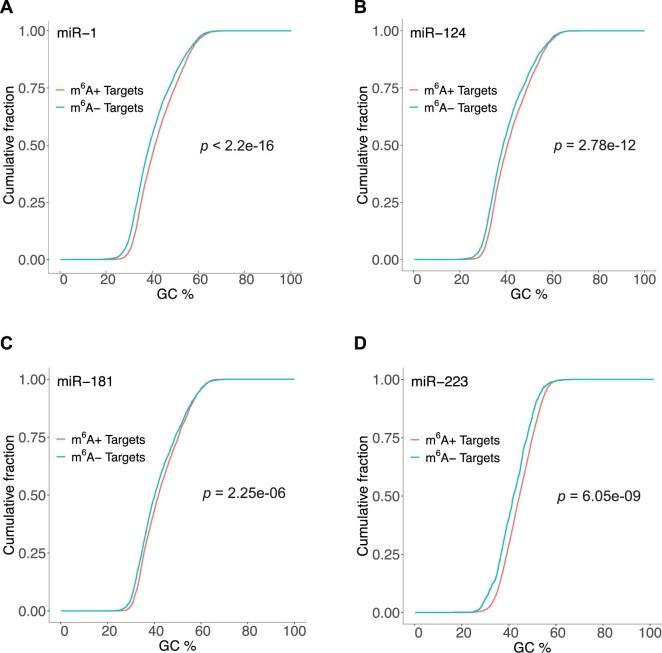
Methylated target 3′-UTRs have higher GC content. Comparison of cumulative distributions of GC% between m^6^A+ targets (red) and m^6^A− targets (green). A significant higher GC% is observed for (**A**) miR-1 targets with a *P*-value <2.2e-16; (**B**) miR-124 targets with a *P*-value of 2.78e-12; (**C**) miR-181 targets with a *P*-value of 2.25e-06; and (**D**) miR-223 targets with a *P*-value of 6.05e-09

We compared the GC content of the 3′-UTR between the m^6^A+ targets and the m^6^A− targets. We found that for each of the four miRNAs, the GC content for the m^6^A+ targets was significantly higher than that for the m^6^A− targets (miR-1: Fig. 3A, *P*-value <2.2e-16; miR-124: Fig. 3B, *P*-value of 2.78e-12; miR-181: Fig. 3C, *P*-value of 2.25e-06; miR-223: Fig. 3D, *P*-vale of 6.05e-09). Because GC pairing is thermodynamically more stable than AU pairing, the finding indicates that m^6^A+ targets are structurally more stable than m^6^A− targets.

**Fig. 4.**
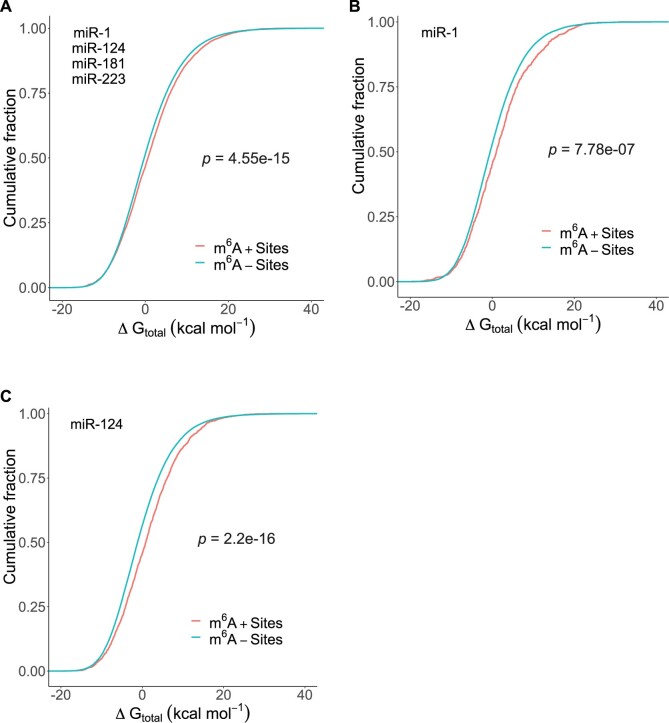
MiRNA-binding sites that can be potentially m^6^A modified are structurally less accessible based on free energies of unmodified nucleotides. Comparison of cumulative distributions of Δ*G*_total_ for m^6^A+ binding sites (red) and m^6^A− binding sites (blue), for (**A**) miR-1, miR-124, miR-181 and miR-223 pooled together, with a *P*-value of 4.55e-15; (**B**) miR-1, with a *P*-value of 7.78e-07; and (**C**) miR-124, with a *P*-value of 2.2e-16

To further investigate potential interplay between m^6^A and the target secondary structure, we performed a target accessibility analysis for all predicted binding sites for miR-1, miR-124, miR-181 and miR-223. We computed Δ*G*_total_, the total energy change of miRNA: target hybridization, which is an energetic measure of target structural accessibility (Long *et al.*, 2007). For the binding sites pooled together for all four miRNAs, Δ*G*_total_ was significantly higher for m^6^A+ sites than m^6^A− binding sites (Fig. 4A; *P*-value of 4.55e-15). For each individual miRNA, a higher Δ*G*_total_ was observed for m^6^A+ sites in comparison to m^6^A− sites. Two examples are shown for miR-1 (Fig. 4B, *P*-value of 7.78e-07) and miR-124 (Fig. 4C; *P*-value of 2.2e-16). A higher Δ*G*_total_ indicates lower accessibility. For target structure predictions and free energy calculation, the RNA thermodynamics in the computation are for unmodified nucleotides. Thus, the observation on accessibility is for unmodified targets. The finding indicates that a miRNA-binding site that can be m^6^A modified is structurally less accessible in the absence of methylation. We did not observe such an effect with statistical significance for the CLASH data. This could be due to differences in cellular environments for different experimental systems. For RNA/RNA interaction, e.g. when both molecules are present at high concentrations, the equilibrium would favor hybridization, regardless of local structural accessibility. In contrast, difference in conservation is an evolutionary signal that does not depend on experimental conditions.

### 3.5 A secondary structure-based model

Two previous studies have reported enhancement or suppression of miRNA targeting through alteration of a local target secondary structure upon binding by RNA-binding proteins (RBPs) (Kedde *et al.*, 2010; Xue *et al.*, 2013). In our study context, enhanced target site accessibility and miRNA targeting could possibly be facilitated through the binding of a m^6^A reader protein.

The effects of m^6^A on RNA structure have been studied by RNA structure probing using RNases V1 and nuclease S1 (Liu *et al.*, 2015), and by high-throughput structure probing using icSHAPE (Spitale *et al.*, 2015). Both studies reported that m^6^A can alter the base-pairing status of neighboring nucleotides from paired to unpaired, thereby increasing the local structural accessibility. It has been reported that modified adenosines including m^6^A reduce stability of RNA duplexes (Kierzek and Kierzek, 2003). In a biochemical study, it was reported that N6 methylation within a helical region can have destabilizing effect, whereas methylation of an unpaired base can increase the stability of single base stacking (Roost *et al.*, 2015). These findings suggest that m^6^A alone could be sufficient for modulation of regulation.

**Fig. 5.**
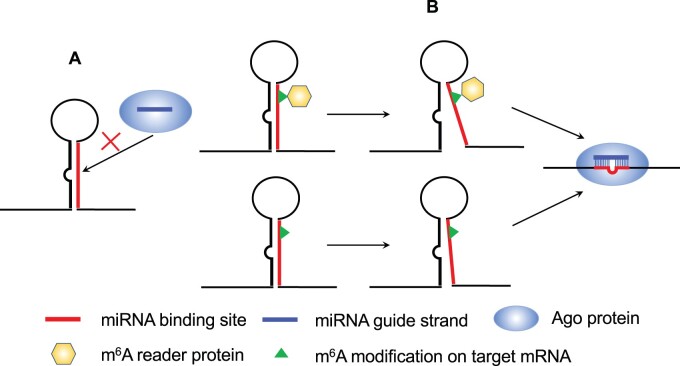
A proposed model of m^6^A function in miRNA targeting. m^6^A alters the local secondary structure of the target RNA to increase accessibility for binding by AGO. (**A**) In the absence of m^6^A, the local target structure at the miRNA-binding site is structurally inaccessible; (**B**) m^6^A is recognized and bound by an m^6^A reader, and this interaction opens the local structure (top); m^6^A alone is sufficient to significantly weaken the local target secondary structure (bottom), resulting in a structurally accessible miRNA-binding site facilitating AGO binding and miRNA-mediated regulation

To answer the question of why regulation is enhanced for modified targets, given that miRNA-binding sites that can be modified are less accessible before methylation than unmodified sites, we propose two specific secondary structure-based mechanistic models (Fig. 5). In the first model, m^6^A within the miRNA-binding site is recognized and bound by an m^6^A reader protein(s), leading to opening of the local target secondary structure, which facilitates binding by AGO for enhanced regulation. In the second m^6^A reader-independent model, the presence of m^6^A alone is sufficient for alteration of the local structure, leading to increased site accessibility for Ago binding.

## 4 Discussions

In this study to explore the potential interaction between m^6^A modification and gene regulation by miRNAs, we performed comprehensive analyses of high-throughput data on m^6^A in human and mouse transcriptome, miRNA target binding and regulation. There are two possible outcomes of miRNA-mediated gene regulation: mRNA degradation or translational inhibition. The levels of mRNAs in an experimental system can be easily measured by microarrays or RNAseq. However, measurements of levels of large number of proteins are much more difficult and can be very costly. Our analyses are thus limited to available high throughout mRNA data. On the other hand, it has been reported that miRNAs predominantly act to decrease target mRNA levels, and destabilization of target mRNAs is the predominant reason for reduced protein output (Guo *et al.*, 2010).

We found that the level of regulation is significantly higher when m^6^A is present on target mRNAs. The evolutionary conservation for miRNA-binding sites with m^6^A modification is significantly higher than that for miRNA-binding sites without modification. These findings strongly indicate the functional significance of m^6^A modification in miRNA-mediated gene regulation.

The RNA modification database used in this study compiles experimental data from different experiments (Sun *et al.*, 2016). Differences in cell lines and techniques in these experiments can present different m^6^A profiles, as m^6^A modification is cell-type specific. For these reasons, some of the m^6^A+ targets may not be methylated in the specific cell line for the microarray study (Baek *et al.*, 2008). It is impossible to determine which of the m^6^A+ targets are methylated and which are not. The observed effects of the enhanced miRNA regulation by methylation could be diluted by the inclusion of targets that are not methylated. In other words, the true regulatory effects by m^6^A could be greater than what were observed here.

To explore a model-based explanation of our findings, we performed a target site accessibility analysis based on a target secondary structure prediction and miRNA-target hybridization modeling with RNA thermodynamic parameters for unmodified nucleotides. While modeling the secondary structure of RNAs with modified nucleotides would be ideal, complete thermodynamic parameters for chemical modifications including m^6^A are not available. We found that miRNA-binding sites that can be potentially m^6^A modified are significantly less accessible in the absence of methylation than those that do not have potential methylation sites. This is in sync with the finding that m^6^A+ targets are structurally more stable than m^6^A− targets, as indicated by their higher GC content.

Our findings and known role of m^6^A in destabilizing RNA structure led to a proposed model in which m^6^A can alter local target structure to increase accessibility for binding by AGO, leading to enhanced regulation. Specifically, we propose an m^6^A reader-dependent model and an m^6^A reader-independent model (Fig. 5). Our model is limited to modification within miRNA-binding sites. Because long-range base-pairing interaction is possible in mRNAs, we speculate that in some cases modification outside miRNA-binding sites can also enhance regulation if the modification unpairs base-pairs involving nucleotides within a miRNA-binding site.

In one recent computational study, spatial correlation among m^6^A, AGO binding and binding of RBPs led to a proposed model for a three way interplay mediated through alteration of the target RNA secondary structure (Das Mandal and Ray, 2021). For two of the four modes in the model, methylation facilitated target binding by miRNAs. In another recent study with a focus on RBPs and miRNA targeting, most RBPs were found to enhance miRNA targeting by increasing target site accessibility (Kim *et al.*, 2021). For miRNA targeting, the importance of target structure and binding site accessibility was established over 14 years ago (Long *et al.*, 2007). The findings from this study and the two recent studies signal the emergence of target structure as a common theme in our expanding understanding of miRNA-mediated gene regulation.

We hope our key finding of enhanced miRNA targeting by m^6^A and the two proposed models can be directly tested through properly designed experiments. To this end, m^6^A writer knockouts, nucleotide mutagenesis at the modification site that preserves base-pairing status, and an assay for miRNA regulatory activity can be useful tools. Local base-pairing status can be assessed either by experimental structure probing or by computational prediction (Ding *et al.*, 2004; Ding and Lawrence, 2003). For a measurement of local structural accessibility, Δ*G*_total_ is available from STarMir (Rennie *et al.*, 2014).
